# Layer-by-Layer Fabrication of Hydrogel Microsystems for Controlled Drug Delivery From Untethered Microrobots

**DOI:** 10.3389/fbioe.2021.692648

**Published:** 2021-10-13

**Authors:** Roberto Bernasconi, Fabio Pizzetti, Arianna Rossetti, Brendan Butler, Marinella Levi, Salvador Pané, Filippo Rossi, Luca Magagnin

**Affiliations:** ^1^ Department of Chemistry, Materials and Chemical Engineering“Giulio Natta”, Politecnico di Milano, Milano, Italy; ^2^ Multi-Scale Robotics Laboratory, Institute of Robotics and Intelligent Systems, ETH Zurich, Zurich, Switzerland

**Keywords:** microrobots, 3D printed, drug delivery, hydrogels, layer-by-layer

## Abstract

Targeted drug delivery from untethered microrobots is a topic of major interest in current biomedical research. The possibility to load smart materials able to administer active principles on remotely *in vivo* guidable microdevices constitutes one of the most attractive opportunities to overcome the drawbacks of classical untargeted delivery methodologies. Hydrogels, in particular, are ideal candidates as drug-carrying materials due to their biocompatibility, low cost, and ease of manufacturing. On the other hand, these polymers suffer from poor control over release rate and overall released amount. Starting from these premises, the present article demonstrates the possibility to tune the release of hydrogels applied on magnetically steerable microrobots by fabricating microsystems *via* layer-by-layer self-assembly. By doing this, the diffusion of chemicals from the hydrogel layers to the external environment can be optimized and the phenomenon of burst release can be strongly limited. The microrobotic platforms employed to transport the hydrogel active material are fabricated by employing 3D printing in combination with wet metallization and present a gold layer on their surface to enhance biocompatibility. The maneuverability of microdevices coated with both thin and thick multilayers is investigated, individuating optimized parameters for efficient actuation.

## Introduction

In the last few decades, the medical approach toward the administration of drugs has been characterized by radical improvements. Together with the continuous development of new active principles, research has also been focused on the improvement of delivery techniques for already existing formulations (Alvarez-Lorenzo and Concheiro, 2014; Holowka and Bhatia, 2014; Wang et al., 2016). Indeed, usage optimization is a matter of major relevance when considering the problems generally related to conventional drug administration: misuse (either self-conscious or related to difficult posology), presence of negative side effects and induced drug resistance for cancer (Vasan et al., 2019) and microorganisms (Sekyere and Asante, 2018). These drawbacks can be limited by developing administration routes independent of blood-mediated distribution (Gibaldi et al., 1971) or transdermal diffusion (Ita, 2014). Considering the practical importance of the topic, many such innovative smart administration methodologies have been proposed, from liposomes (Pattni et al.2015, Lee and Thompson 2017) to functional nanoparticles (Vangijzegem et al., 2019, Ghitman et al., 2020, Manzano and Vallet‐Regí 2020) and DNA functional nanostructures (Hu et al., 2018) or polymeric microneedle arrays (Vecchione et al., 2014; Ruggiero et al., 2018).

The most interesting solution for targeted delivery, however, is probably the direct transportation of the drug exclusively toward the affected area by means of untethered microdevices able to *in vivo* navigate the body (Nelson et al., 2010; Chen et al., 2017; Palagi and Fischer, 2018). Micromotors capable of remote propulsion and carrying an active material able to store and release the drug have been described in the literature in a wide range of dimensions (Fusco et al., 2014; Li et al., 2016; Erkoc et al., 2019; Jang et al., 2019), according to the target organ for which they are intended [e.g., hundreds of micrometers for the gastrointestinal apparatus (Bernasconi et al., 2020) or few micrometers for blood vessels (Park et al., 2019)]. Biomedical microrobots are, in the vast majority of the cases, actuated by applying controlled magnetic fields (Xu et al., 2015; Sitti, 2017; Yang and Zhang, 2020; Ebrahimi et al., 2021). These constitute an ideal choice for remote *in vivo* guiding since they are noninvasive toward biological tissues, are highly precise and do not require *in-situ* energy sources on the devices [in contrast, for example, to chemical propulsion (Zhu et al., 2015)].

From the drug release point of view, the choice of proper active material is crucial for effective delivery. Devices described in the past were based on a wealth of polymeric materials, both synthetic and natural: hydrogels (Hoare and Kohane 2008, Li and Mooney 2016) and chitosan (Giri et al., 2012) as well as conducting polymers (Hathout et al., 2018; Shah et al., 2018). In addition, the recent development of smart stimuli-responsive materials further expanded drug targeting efficiency by subordinating the release to the presence of an external trigger (Wang et al., 2014; Guragain et al., 2015). In this way, researchers developed microdevices that are able to reach specific organs and administer drugs on demand thanks to pH (Li et al., 2016; Chen et al., 2020), temperature (Qiu et al., 2014) and light (Bozuyuk et al., 2018) variations or ultrasound application (Darmawan et al., 2020). Among the active materials described in the literature, hydrogels are well known for their biocompatibility, efficiency in drug loading/release, and ease of manufacturing (Hoffman, 2012; Narayanaswamy and Torchilin, 2019). In particular, a very interesting aspect of this kind of product is the possibility to tune its properties (degradation rate, swelling, release of drugs, and mechanical resistance), according to the application, just by opportunely choosing reactants and production environment conditions. Starting from their remarkable characteristics such as tunable physical, biological, and chemical properties, high biocompatibility, versatility in production and application, and a wide range of applications (regenerative medicine, drug and gene delivery, scaffolding for cells, stem cell and cancer research, and advanced therapies), it is not surprising that they are emerging as the most promising materials in the biomedical field (Pulat and Uğurlu, 2016; Akalin and Pulat, 2020). In this context, remotely guidable microdevices, coated with hydrogel materials have been proposed (Li et al., 2016; Lee et al., 2018). Hydrogels can also be easily functionalized by linking the drug to the hydrogel with cleavable bonds so that it is released only in specific conditions (Mauri et al., 2017; Bernasconi et al., 2021).

In the present work, we propose a methodology to control drug delivery from magnetically guided microdevices by encapsulating the alginate drug-releasing material with a hydrogel microsystem fabricated via a layer-by-layer approach (Wang and Newby, 2018). The main aim is, therefore, to extend and control the diffusion path of the drug out from the gel matrix by coating the surface of the alginate hydrogel with an alternating sequence of layers of oppositely charged polyelectrolytes in order to create a protective barrier that tunes the release rate. With respect to our previous work (Bernasconi et al., 2021), where the drug-releasing hydrogel was chemically functionalized to release under certain pH conditions, the approach presented in the present article is less specific and more versatile. The release cannot be triggered, but the multilayered microsystems described hereby present important advantages: they are less costly, they do not need specific synthesis routes (making them ideal for a wide variety of drugs), and they do not chemically alter the drug upon release. Following our previous work (Bernasconi et al., 2021), the geometry of the devices and alginate coating procedure were optimized. The best pair of oppositely charged materials to be used was determined. At last, release and magnetic actuation tests were carried out to certify the efficiency and practicality of the developed approach.

## Experimental Methods

### Microdevice 3D Printing and Metallization

Microdevices were 3D printed by employing micro stereolithography and wet-metallized following a procedure analogous to the one detailed in our previous work (Bernasconi et al., 2019). Briefly, devices presenting the optimized geometry were designed using SolidWorks (Dassault Systèmes, France), optimized with Nauta+ (DWS, Italy), sliced with Fictor (DWS, Italy), printed using a model 028 J Plus stereolithography setup (DWS, Italy), and finally postcured by exposition to UV radiation for 30 min. At the end of the printing process, samples were removed from the printing supports and moved to a hybrid electroless/electrolytic metallization step. The surface of the devices was cleaned, etched, activated, and coated with the first layer of electroless copper following the process described in the literature (Bernasconi et al., 2019). Subsequently, a layer of electrolytic CoNiP was applied. After the application of the CoNiP layer, the process differed from the cited literature reference. In particular, the surface of the devices was coated with electrolytic gold employing the barrel plating approach. In detail, pure gold was electrodeposited using a SG-Au 340 bath (SG Galvanobedarf GmbH) in the following conditions: 5 mA/ cm^2^, moderate stirring, and 50°C. At the end of the process, Au-coated devices were washed and dried with nitrogen.

### Layer-By-Layer Coating Process

To coat the devices with hydrogel layers, they were dipped sequentially into the polymer aqueous solutions and then in the physical crosslinker in order to deposit the 3D networks. With the goal of obtaining a homogeneous coating of the device, a small plastic structure was utilized together with a very thin nylon wire in order to hang the device, preventing it from touching any part of the beakers containing the solutions. Such plastic structure is schematized in Supplementary figure S2. Initially, a central alginate layer was formed by immersion in a 2 % wt. alginate aqueous solution (figure 6.17 a). This step was performed inside an ultrasonic bath in order to favor the penetration of the alginate chains inside the scaffold matrix. This first layer was immersed in the crosslinker aqueous solution (2 % wt. CaCl_2_) to obtain the first hydrogel layer. This first alginate layer was further coated with multilayers of either chitosan/alginate (CHT/ALG) or poly(allylamine) hydrochloride/alginate (PAH/ALG). In the first case, chitosan was dissolved in a sodium phosphate buffer solution (pH 6) at a concentration of 0.4% w/v. To form the chitosan layer, alginate-coated devices were immersed in the chitosan solution for 10 min, removed, and let sit for 2 min. Then, the structure was immersed in the alginate solution for 2 min, removed, and gelated in 2 % wt. CaCl_2_. By following this procedure, a single CHT/ALG bilayer was formed on the surface. The procedure was repeated to form further bilayers. In the case of PAH, the same procedure was followed by substituting the chitosan solution with a 0.4% w/v aqueous solution of PAH. In summary, alginate (used as polyanion) was coated with polycations (ALG or PAH) and then again with alginate to form CHT/ALG and PAH/ALG samples with 1 bilayer, respectively. Then, continuing this procedure, we were able to obtain two and three bilayers.

### Device Characterization

A Zeiss EVO 50 SEM setup was employed. The same was equipped with an Oxford Instruments Model 7060 EDS module. The magnetic properties of the devices were studied using a Princeton Measurement Corp. MicroMag 3900 vibrating sample magnetometer (VSM) setup. Surface roughness was evaluated by means of a UBM Microfocus laser profilometer.

### *InVitro* Drug Delivery and Mathematical Modeling

The drug release mechanism was investigated in simulated physiological conditions: at 37°C and 5% CO_2_, in a phosphate-buffered saline solution (PBS, pH 7.4). Rhodamine B (RhB), a widely used model molecule, was used to verify drug release performances. RhB was loaded in the first alginate layer, following the same procedure described above with the addition of RhB. So, the device was dipped in alginate RhB (1 mg/ ml) solution. Then, to avoid the loss of RhB during the other dipping steps, all the other solutions were prepared in the presence of RhB at the same concentration. This approach can guarantee on one side 100% of drug loading and on the other to avoid the uncontrolled loss of the drug content. Then, each device was placed in excess of PBS (2.5 ml), and aliquots were collected at defined time points, replacing them with an equal volume of fresh solution, in order to preserve the diffusion regime among the device and the release environment. Percentages of released RhB were then measured by UV spectroscopy at a specific wavelength (570 nm). Drug diffusion mechanism can be described as a 1-dimensional model of the second Fick law where the device geometry is a cylinder and the material flux mainly takes place at the PBS/hydrogel surface. Eq. 1 shows these considerations, indicating r as the characteristic radius for the mass transport phenomenon. The following mass balance equations are written considering the variation of the mean drug concentration within the hydrogel (C_G_) related to the volume of solution (V_S_), the mean drug concentration in the outer solution (C_S_), the total volume (V_G_), the drug present inside the matrix (m_G_), and the exchange interfacial surface (S_exc_), which represents the boundary surface between the device and the surrounding solution (which, simplifying, can be here considered as being only the side surface). According to these expressions, the boundary conditions are defined describing the profile symmetry at the center of the polymeric cylinder, with respect to the radial axis of the cylinder (Eq. 5) and the equivalence between the material diffusive fluxes at the PBS/hydrogel surface (Eq. 6).

$$\frac{\partial C_{G}}{\partial t}=D\cdot \frac{1}{r^{2}}\cdot \frac{\partial }{\partial r}\cdot \left(r^{2}\cdot \frac{\partial C_{G}}{\partial r}\right)$$ (1)

$$V_{S}\frac{\partial C_{G}}{\partial t}=k_{C}\cdot S_{exc}\cdot \left(C_{G}-C_{S}\right)$$ (2)

$$C_{S}\left(t=0\right)=0$$ (3)

$$C_{G}\left(t=0\right)=C_{G,0}=\frac{m_{G,0}}{V_{G}}$$ (4)

$${\frac{\partial C_{G}}{\partial r}|}_{r=0}=0$$ (5)

$${-D\cdot \frac{\partial C_{G}}{\partial r}|}_{r=R}=k_{C}\cdot \left(C_{G}-C_{S}\right)$$ (6)

This mathematical model allowed estimating the diffusion coefficient (D) of RhB. Sherwood number obtained by means of penetration theory allowed the computation of the mass transfer coefficient k_C_ (Eq. 7).

$$Sh=1=\frac{k_{C}\cdot 2r}{D}$$ (7)

### Actuation Tests

The magnetic actuability of the devices was tested by employing the OctoMag magnetic manipulation setup (Kummer et al., 2010) to impart a rolling motion. Microrobots were permanently magnetized along the direction perpendicular to their axis by placing them on a NdFeB magnet. Then, a rotating magnetic field characterized by varying intensity and frequency (τ) was applied. The magnetization M present in the device continuously aligned with the external field B, generating a torque according to the following equation:

$$\vec{T}=v\;\vec{B}\;\times \;\vec{M}$$ (8)

*where v* is the volume of magnetic material present in the device, *M* is its magnetization, and *B* is the field acting on the device. Thanks to the rotating field, *B* continuously rotated, thus generating a continuous rotation of the device. Analogously to a wheel, the microrobot moved forward when placed on a solid surface. Uncoated and hydrogel-coated devices were actuated inside a water-filled glass basin to avoid hydrogel desiccation. Their motion was tracked and their speed was determined using the software Tracker.

## Results and Discussion

### Shape Optimization and Production Route

**FIGURE 1:**
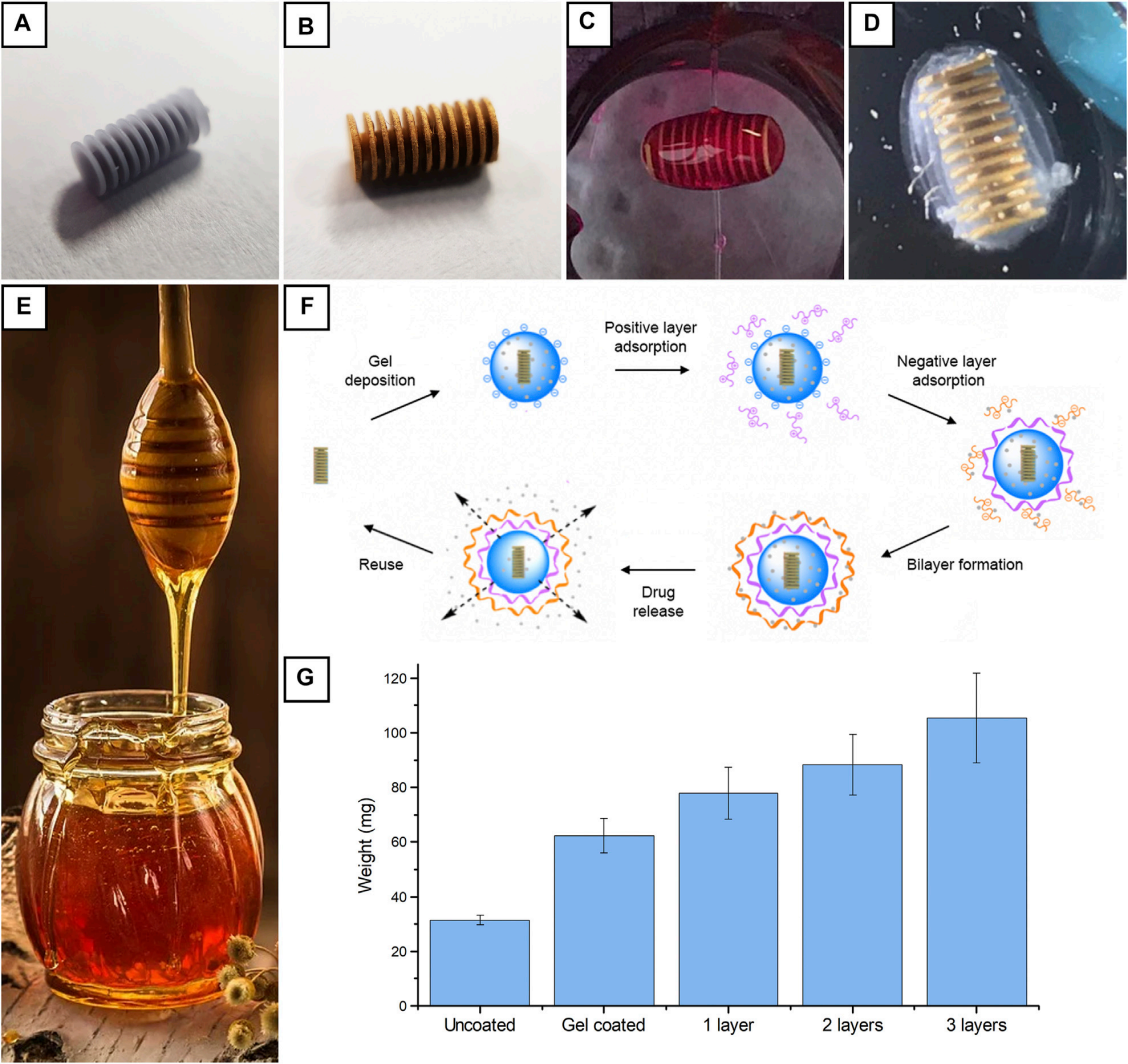
Visual appearance of a device after 3D printing and support removal **(A)**; visual appearance after Cu, CoNiP, and Au deposition **(B)**; device coated with a single layer of RhB-loaded alginate hydrogel **(C)**; device coated with a hydrogel multilayer **(D)**; a honey dipper **(E)**; hydrogel coating procedure starting from the gold-coated device **(F)**; weights for uncoated, alginate-coated, and multilayer-coated devices **(G)**.

In our recent work, we employed scaffold-like architectures to support and transport hydrogels (Bernasconi et al., 2021). The choice of using a scaffold was motivated by the possibility to load a high amount of hydrogel inside the device, exploiting its porosity. In the present work, geometry was further optimized taking inspiration from the honey dipper, a tool employed for the domestic manipulation of highly viscous fluids, like honey (Figure 1E). Its shape, which is composed of stacked disks, is ideal to capture and hold thick fluids. Since the hydrogel is a viscous fluid prior to gelation, the same concept can be transferred to suitably designed untethered microdevices. Small honey dippers, whose dimensions are detailed in Supplementary figure S1, were designed and 3D printed. Features were kept in the few millimeters to hundred micrometers dimensional range. This choice was motivated by the possible use of the devices in the gastrointestinal apparatus, whose tracts present characteristic dimensions in the few centimeters range (e.g., 5 cm of diameter in the colon and 3.5 cm diameter in the ileum). A typical batch of devices was composed of 10–15 units and it was successfully printed in a 30-min timespan.

After printing, devices were removed from their printing supports (Figure 1A) and metallized. Electroless copper was first applied on the surface of the microdevices to make them conductive (Bernasconi et al., 2019). Subsequently, the barrel plating approach was employed to apply CoNiP from electrolytic plating. CoNiP ferromagnetic properties were exploited to make the devices actuable via magnetic field application. 400 nm of Cu was applied via immersion in a formaldehyde-free copper electroless bath, while 5 μm of CoNiP was deposited using the barrel plating approach. The latter allows the deposition of electrolytic layers on small-scale devices by simply placing them inside a polarized metallic basket immersed in the desired electrolyte. As previously observed, the nonideal contact between the devices and the basket requires the introduction of a correction coefficient ψ in the Faraday law for electrolysis (Eq. 9) that describes the amount of material plated with respect to the total quantity of electrons employed.

$$m=\;\frac{Mq}{ZF}\;\eta \;\psi$$ (9)

where *m* is the total mass deposited, *M* is the molar mass of the metal, *q* is the total electric charge, *Z* is the valence of the ions reduced, *F* is the Faraday constant, η is the cathodic efficiency of the deposition reaction, and ψ is the apparent efficiency of the barrel plating process. η and ψ for CoNiP deposition are equal to 0.42 and 0.25, respectively.

After CoNiP plating, the surface of the devices was coated with gold (Figure 1B) to make them biocompatible. In our previous works (Bernasconi et al., 2018; Bernasconi et al., 2021), gold was applied via galvanic displacement. This technique, however, does not allow complete control over the thickness of the gold layer, since galvanic displacement is an intrinsically self-limiting reaction. Indeed, deposition tends to stop when the surface is completely coated with the metal, thus limiting the obtainable thickness to few hundred nanometers. In contrast, it is attractive to coat the devices with some micrometers of Au. In this way, the chemical resistance of the devices is strongly enhanced and the number of reuse cycles can be increased without losing biocompatibility. Consequently, barrel plating from a cyanide-free electrolyte was used to deposit gold on the devices. Since this process was carried out for the first time, η and ψ were determined. The cathodic efficiency η of the deposition process was evaluated by depositing gold on a planar copper substrate. By doing this, no barrel was used and ψ was assumed to be equal to 1. By comparing the mass of metal deposited with the charge employed, cathodic efficiency η resulted to be equal to 0.57. ψ was evaluated by weighting the devices before and after deposition in the barrel, considering the cathodic efficiency previously calculated and comparing the result with the total charge employed. ψ resulted to be equal to 0.28.

After metallization, devices were coated with hydrogel multilayers to delay the release of the drug loaded into the hydrogel carrier (Figure 1D). We already demonstrated that the deposition of a layer of sodium alginate hydrogel over the device *via* dip coating (Bernasconi et al., 2021) was a valid solution to introduce a drug delivery function (Figure 1C). Such layer was loaded with physically trapped RhB and its release behavior was investigated. The study evidenced that RhB immediately started to diffuse out from the hydrogel, and in 1–2 h, the layer was completely depleted. Consequently, additional layers were deposited on the initial alginate to tune the diffusion of RhB out of the device. In this way, the rate of drug release during transportation toward the target region was reduced and the initial burst release was limited. This approach was investigated because it is a simpler and costless technique with respect to more sophisticated techniques such as hydrogel functionalization (Bernasconi et al., 2021). Consequently, it allows fast and cost-effective fabrication of optimized drug-releasing devices based on hydrogels.

Multilayers were deposited by dip coating using a layer-by-layer (LbL) approach (Figure 1F). Following this strategy, layers of polymers with different charges can be used: we used alginate as an anionic polymer because it showed good adhesion to the microrobots (Bernasconi et al., 2021) and chitosan or poly(allylamine) (chemical structures are reported in Supplementary figures S6–S8) as cationic polymer. Initially, alginate was applied on the devices. Then, a layer of positively charged hydrogel (either chitosan or poly(allylamine) hydrochloride) was electrostatically adsorbed on the surface of the alginate by means of immersion. Subsequently, negatively charged alginate was electrostatically adsorbed on the positive layer. Samples coated with chitosan/alginate bilayers were identified as CHT/ALG, while samples coated with poly(allylamine) hydrochloride/alginate bilayers were identified as PAH/ALG. On some samples, the adsorption process was repeated to deposit more than one bilayer. By controlling the number of bilayers, the diffusion path of the drug was significantly increased and the release was tuned. Figure 1F describes the weight increase recorded in the case of CHT/ALG multilayer deposition. As it is evident from the graph, the initial hydrogel coating almost doubled the weight of the device, thus suggesting a good efficiency of the honey dipper structure in retaining the hydrogel inside the device. Subsequent coating cycles easily increased the weight of the device due to the easy buildup of positively and negatively charged layers on the surface of the initial hydrogel coating. In general, the total amount of material present on the device could be easily controlled by varying the number of coating cycles.

At the end of the drug release step, samples can be efficiently reused by removing the hydrogel present on the surface. Since the base of the device is a rigid 3D printed and wet metallized structure, exhaust hydrogel can be removed simply by immersion in a solution containing a weak acid (like citric acid). This was demonstrated by immersing the devices in diluted sodium citrate solutions. The latter dissolves the hydrogel by extracting crosslinking ions (Boontheekul et al., 2005) and its action is strongly concentration-dependent. Supplementary Figures S3, S4 show the reduction in weight of alginate hydrogels when put inside a citrate solution. In particular, alginate samples characterized by two different concentrations 1 % m/v Supplementary figure S3) and 5% m/v (Supplementary figure S4) were exposed to five different citrate concentrations: 1 mM, 5 mM, 10 mM, 50 mM, and 100 mM. Data obtained demonstrate that the speed of degradation increased for higher citrate concentrations. At lower concentrations, there was a slight increase in weight due to swelling of the samples. Indeed, the citrate, in this case, did not destroy immediately polymeric chains but rather relaxed them (increasing the water uptake and so increasing the weight measured). Furthermore, degradation under the influence of both the 50 and 100 mM citrate solution was measured to proceed approximately 6 times slower for a 5 % m/v alginate sample compared to a 1 % m/v sample. This can be attributed to the higher number of crosslinking sites and to the stronger interactions created between the chains, which are more difficult to dissolve. Indicatively, 300 mM sodium citrate solutions were employed to efficiently clean the surface of the devices. After this step, devices were coated again with a new hydrogel layer.

### Device Characterization

**FIGURE 2:**
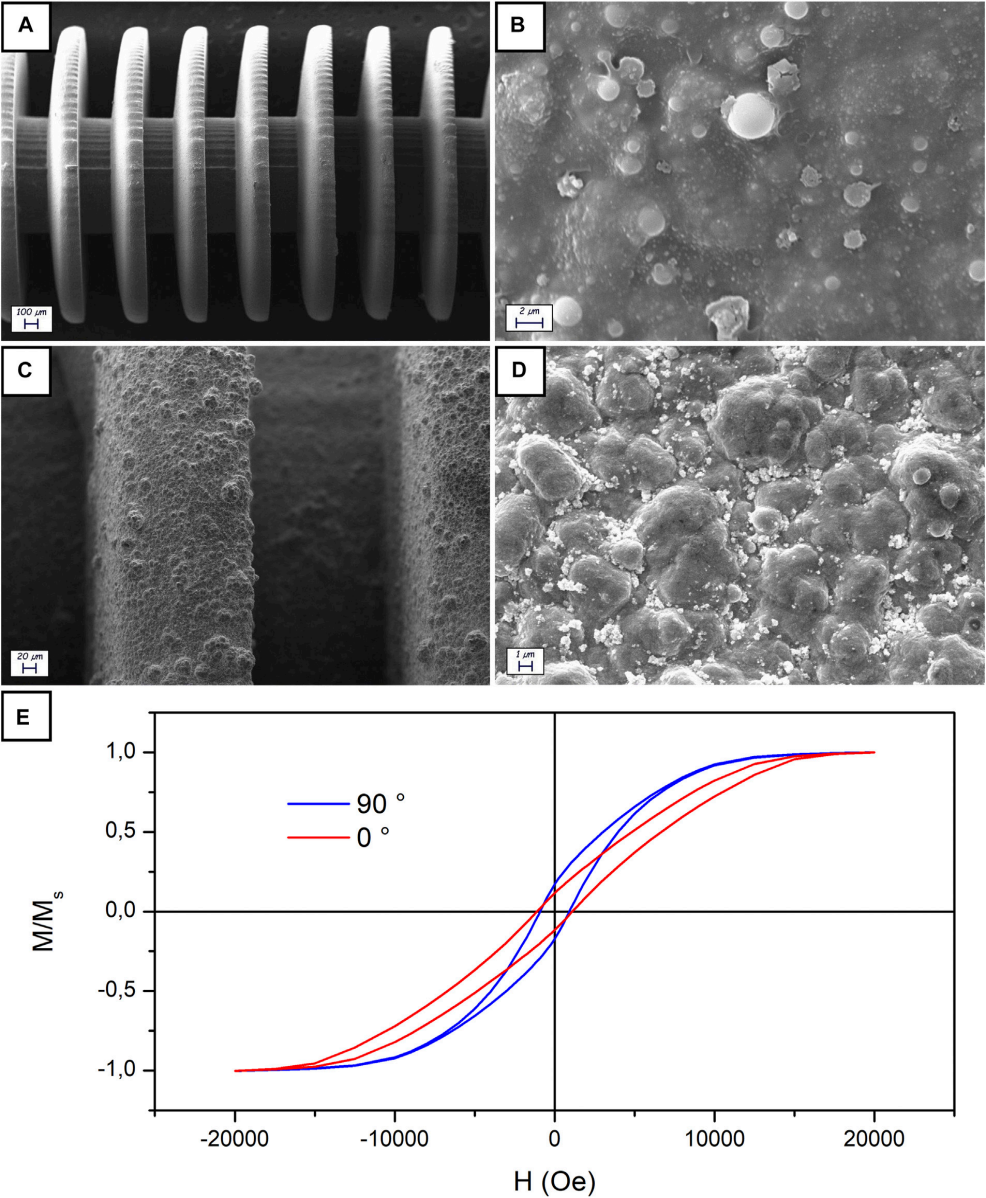
SEM characterization of uncoated **(A, B)** and Cu/CoNiP/Au-coated **(C, D)** devices; VSM of a Cu/CoNiP/Au-coated device **(E)**.

In contrast with electroless Cu and electrolytic CoNiP, which have already been applied and characterized on 3D printed devices (Bernasconi et al., 2019), electrolytic gold was used here for the first time. Consequently, gold-coated devices were characterized to reveal their morphology. Uncoated devices were characterized for comparison, yielding the results visible in Figures 2A,B (at two distinct magnifications). Figure 2A allowed appreciating the dimensional adherence of the printed device to the theoretical dimensions from the 3D model. Figure 2B, on the contrary, allowed clearly observing the microstructure of the stereolithography printed surface. The silicoaluminate filler present inside the resin is visible in the form of spherical particles. After metallization with Cu, CoNiP, and Au, the surface was further characterized (Figures 2C,D).

Figure 2C clearly depicts the structure of the zone between two disks of the device. The metallic layers uniformly covered the surface of the device. Their presence introduced a significant increase in surface roughness, which can be better appreciated in Figure 2D. The typical nodular morphology of electrodeposited Au can be seen in the image. Roughness was evaluated via laser profilometry, yielding a value of R_a_ equal to 321 ± 34 nm. The presence of a relatively high roughness is not detrimental for the scope of the devices, whose functionalities are not affected. On the contrary, roughness could potentially improve hydrogel adhesion to the gold surface. EDS analysis performed on the area visible in Figure 2D gave the result reported in Supplementary figure S5, where almost only Au can be observed on the surface (copper is a spurious signal coming from the sample holder).

Magnetic properties of the devices were investigated using VSM (Figure 2E depicts the result obtained). Since only CoNiP significantly contributed to the magnetic response of the devices, a clear semihard magnetic behavior was observed. Indeed, the magnetic alloy employed was characterized by a Co-rich composition (3.08% wt. P, 84.75% wt. Co and 12.24% wt. Ni, determined via EDS). Coherently with what is visible in Figure 2E, Co-rich and P-poor electrodeposited alloys are normally characterized by hard magnetic characteristics (Park et al., 2002). Values of coercivity varied between 1,124 Oe (along the 0° direction) and 936 Oe (along the 90° direction). Properties are not equal in the two directions (0° refers to the direction parallel to the axis of the device, while 90° is the direction perpendicular to the same axis) due to shape anisotropy.

### Drug Delivery Performances

Once proved that the alginate hydrogel systems can be successfully functionalized with layers of positively charged polymers, RhB release was investigated and compared with the case of solute physically entrapped within the alginate polymeric network. This study was necessary to investigate the benefits related to the use of different layers in our hydrogel systems. Release studies were conducted at 37°C and pH 7.4. The percentage of RhB released was defined as the ratio between the released amount in the aqueous media and the total amount loaded within the polymeric scaffold.

**FIGURE 3:**
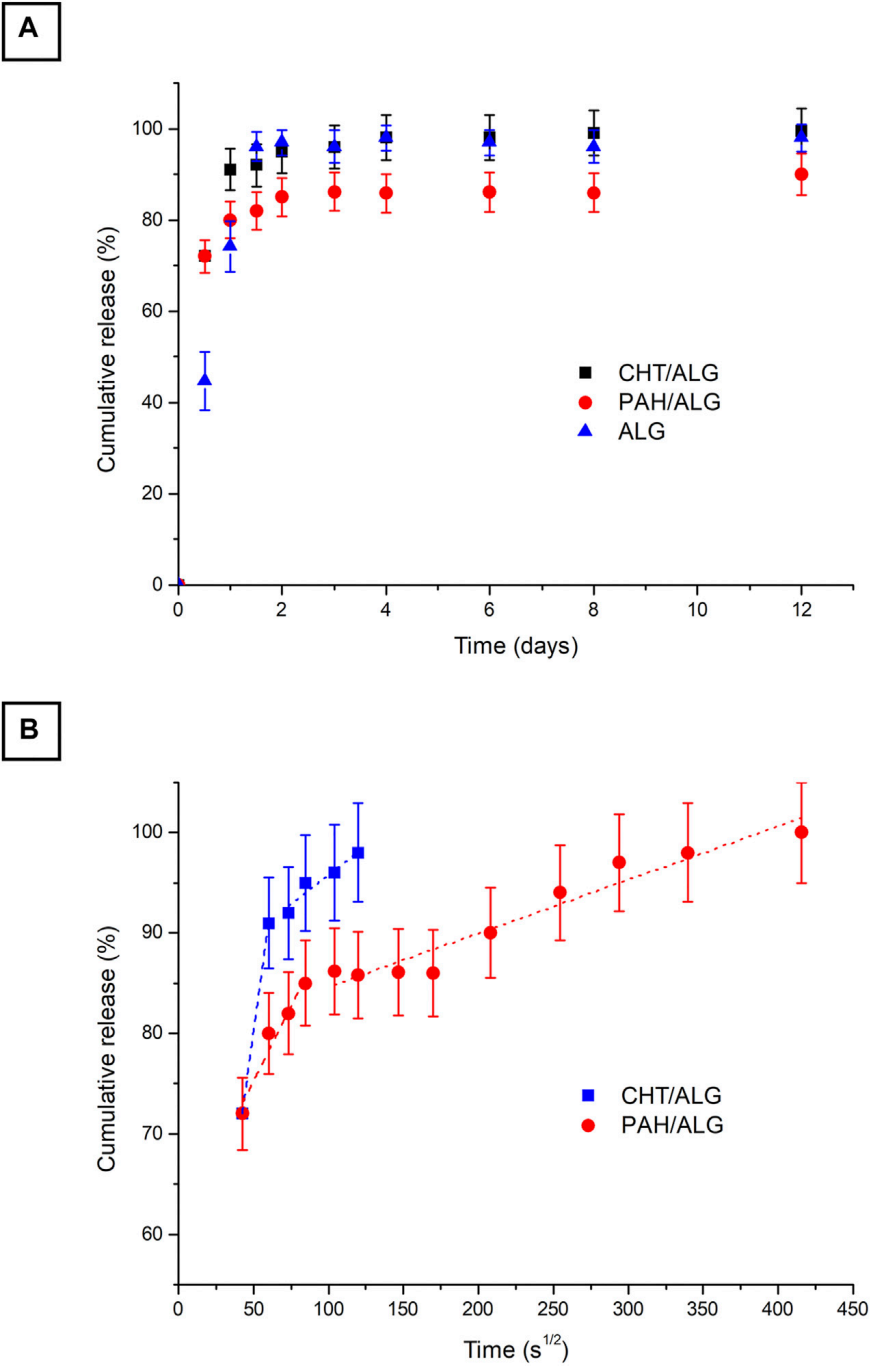
*In vitro* release profile at pH = 7.4 of RhB delivered from ALG (black), CHT/ALG (blue), and PAH/ALG (red) layered hydrogels **(A)**; the slopes of the RhB release from corresponding alginate hydrogels against the square root time **(B)**; the slope of the rhodamine release from hydrogels against the square root time is representative of the Fickian diffusion coefficient of rhodamine in gels (*p* < 0.0001 between all of the groups). The values are calculated as a percentage with respect to the total mass loaded (mean value ± standard deviation is plotted).

In Figure 3A, release profiles associated with CHT/ALG and PAH/ALG were compared with RhB physically entrapped within alginate (ALG) polymeric network. It is well demonstrated that RhB release profile from ALG and CHT/ALG presents the same kinetics trend. PAH/ALG on the other hand showed a slower release rate. There the influence of the system in delivering rhodamine was investigated by plotting release percentage against time square root (Figure 3B). A linear plot is indicative of Fickian diffusion and the y-axis intercept value is an indication of burst release, where it is well known that an ideal controlled release system should present a linear trend during the time and its y-axis intercept should be equal to zero. RhB loaded within alginate hydrogels shows a linear trend only in the first 4 h and then a plateau trend is visible (Bernasconi et al., 2021).

In summary, at the beginning (when RhB-loaded devices are placed in the releasing medium), there is a burst release, which corresponds to a fast discharge of drug driven by the high concentration gradient. Burst release is then followed by a linear release of drug with time, which corresponds to a pure Fickian diffusion and is only driven by the concentration gradient. Finally, the release curve exhibits a plateau trend. After the release mechanism is completed, the remaining drug entrapped within the hydrogel network is released until the complete degradation of the entire network. In our study, pure Fickian diffusion takes place only for up to 3 h for specimens where RhB is entrapped in ALG devices, while it takes 4 h for CHT/ALG and more than 24 h for PAH/ALG.

Mass release data obtained experimentally (Figure 3) were used to estimate the model drug diffusion coefficients. As explained above, the release mechanism could be considered as a pure Fickian diffusion, being concentration driven through alginate hydrogel pores. Table 1 shows the dependence of RhB diffusivity on different device architectures. It is well visible that RhB evidenced no high differences in diffusivity except from PAH/ALG that also exhibited a slower release rate.

**TABLE 1:** Diffusion coefficient of RhB in alginate-based hydrogels.

	Diffusivity (cm^2^/s)
ALG	2.6 10^−4^
CHT/ALG	2.3 10^−4^
PAH/ALG	1.7 10^−4^

As previously said, thanks to their design, devices can be reused at the end of the first drug delivery cycle.

### Magnetic Actuation

In normal conditions, rolling devices move according to Eq. 10. Their motion can be assimilated to wheels, in which the linear speed is proportional to the radius and to the rotation frequency.

v=2πrτ, (10)

where *r* is the external radius of the device and τ is the frequency of the rotating field. There is a direct linear proportionality between the speed and the radius of the device and between the speed and the rotation frequency. This relationship, however, is valid only when the radius is well defined, the external magnetic field continuously generates a constant torque, and no dissipative forces act on the device.

**FIGURE 4:**
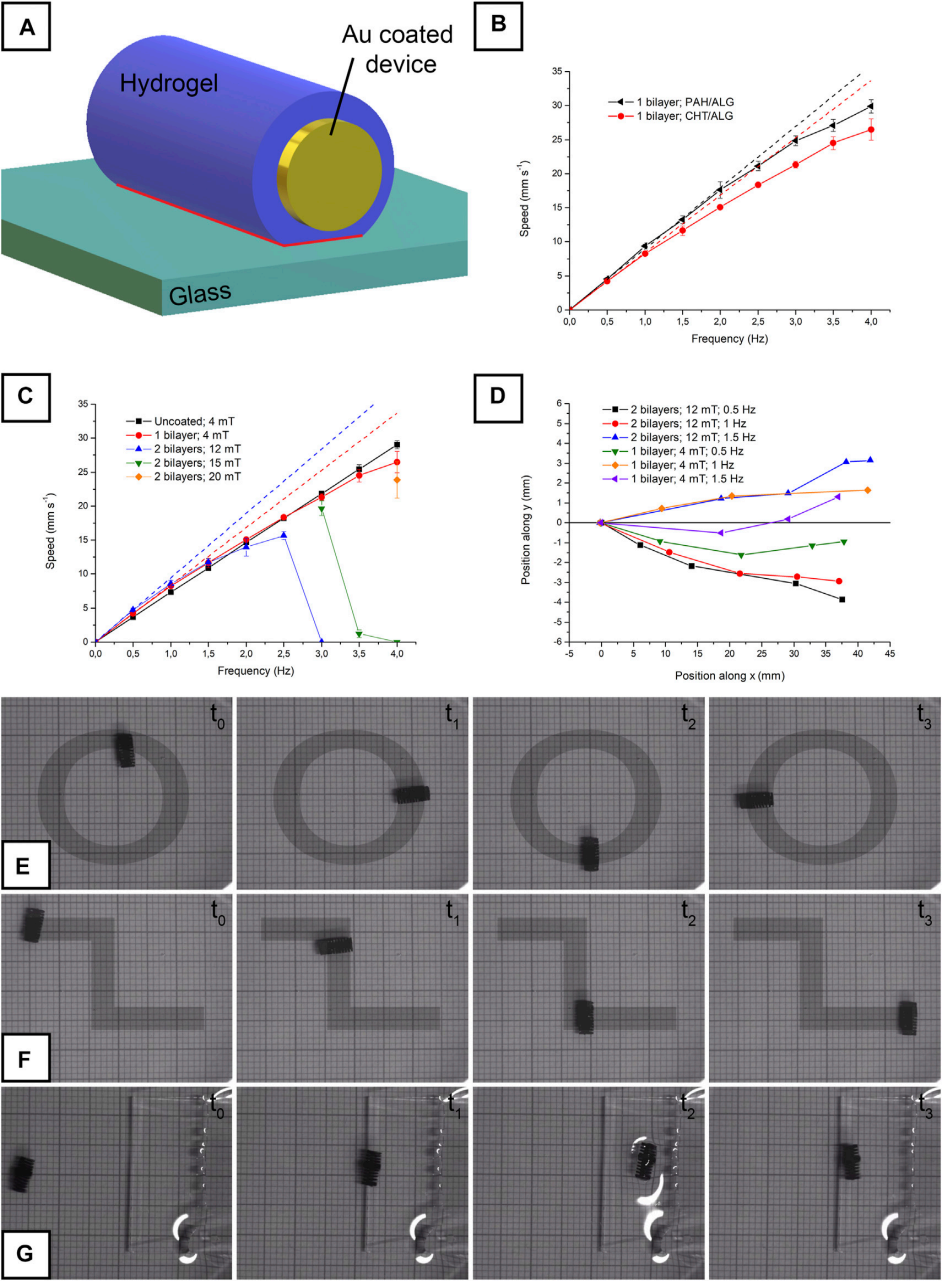
Visual representation of the hydrogel-coated device during actuation, with the contact area between hydrogel and glass highlighted in red **(A)**; speed vs. frequency relationship for single bilayer-coated CHT/ALG and PAH/ALG devices **(B)**; speed vs. frequency relationship for a hydrogel uncoated, for a single bilayer-coated CHT/ALG and for a double bilayer-coated CHT/ALG device **(C)**; deviation from the *y*-axis during actuation for a single bilayer-coated CHT/ALG and for a double bilayer-coated CHT/ALG device **(D)**; circular actuation of a single bilayer-coated CHT/ALG device **(E)**; zig-zag actuation of a double bilayer-coated CHT/ALG device **(F)**; single bilayer-coated CHT/ALG device climbing on a step **(G)**. Panel **(E)**, **(F)**, and **(G)** were acquired using graph paper as background (each square corresponds to 1 mm).

In the case of hydrogel-coated devices, it has already been observed that the presence of the additional layer translates into higher speeds and deviations from linearity (Bernasconi et al., 2021). In the present work, these effects were further investigated and interpreted. Furthermore, the effect of high hydrogel thickness was investigated. In our previous work, hydrogel loading was limited to one layer to maintain the cylindrical shape of the device. Indeed, at high hydrogel thickness, the device loses its cylindrical shape and acquires an ellipsoidal conformation in place (Figure 1D). It is, however, interesting to maximize hydrogel thickness, since this also maximizes drug loading on the device. It is, therefore, crucial to determine whether the devices can be actuated or not in the presence of more than one hydrogel layer. The main challenge in actuating a device coated with a thick hydrogel layer resides in the highly deformable characteristics of the hydrogel itself. As visually schematized in Figure 4A, the hydrogel deforms under the device, resulting in a high contact area (evidenced in red) between the glass substrate and the device itself. This effect introduces a marked nonlinearity in the behavior of the microrobot.

For example, Figure 4B depicts the behavior observed performing a linear actuation (with a 4-mT rotating magnetic field; Supplementary Video S1 visually depicts the typical actuation) on two devices coated with a bilayer of either CHT/ALG or PAH/ALG. It is evident that the speed, coherently with our previous publication (Bernasconi et al., 2021), almost immediately deviated from the linearity expectable from Eq. 10. Such deviation can be realistically attributed to weak adhesive interactions existing between the hydrogel and the glass substrate on the contact area schematized in Figure 4A. Continuously breaking such interactions dissipates energy and lowers the speed of the device. As evidenced in our previous work (Bernasconi et al., 2021), the behavior of the device in the first part of the speed/frequency curve (up to 0.5 Hz) is roughly linear. Consequently, data were linearly fitted to clearly evidence the deviation at higher frequencies (Figure 4B). The radius of the device was extrapolated from the fitting using Eq. 10. The slope of the fitted curves visible in Figure 4B corresponds to the *2πr* section of the equation, thus allowing radius estimation. The radius of the hydrogel-free device resulted to be equal to 1,157 μm, which is very close to the theoretical value of 1,250 µm. In the case of CHT/ALG and PAH/ALG, the radius resulted to be equal to 1,341 μm and 1,430 µm respectively. Consequently, CHT/ALG resulted to be covered by a 184-µm-thick hydrogel layer, while PAH/ALG resulted to be covered by a 272-µm-thick hydrogel layer. These values, however, are not representative of the thickness of the hydrogel in undeformed conditions, since the weight of the device itself squeezes the hydrogel present under the device. This decreases the apparent hydrogel thickness (Figure 4A).

When the thickness of the hydrogel was increased to two bilayers, the behavior of the device significantly changed. Indeed, it was impossible to move the device at any frequency by applying a 4-mT field. Reasonably, adhesive interactions existing between the hydrogel and the glass prevented its detachment from the glass itself. Since the torque applied is proportional to the intensity of B (Eq. 8), it can be inferred that the magnetic field applied was not enough to apply the torque required to start the motion. Consequently, magnetic field strength was increased to 12 mT and the devices moved under these conditions. In particular, they followed the highly nonlinear behavior observable in Figure 4C. Speed was found to monotonically increase up to 2.5 Hz and then it decreased to 0 at 3 Hz. It is well known that rotating magnetic microrobots suffer, at excessive rotation frequencies, the so-called step-out phenomenon (Mahoney et al., 2014). At high rotation speeds, the magnetization of the device is no longer able to follow the frequency of the field. As a consequence, the speed abruptly falls to almost zero. Step-out frequency is influenced by the viscosity of the environment, with higher viscosities generating premature step-out. Something similar takes place in the situation described in the present study for 2 bilayer-coated microdevices. The high adhesion present between the hydrogel and the glass dramatically decreases step-out frequency.

Step-out also depends on the intensity of the applied rotating field, since torque is higher at higher B (Eq. 8). In fact, devices started to move again when the field strength was increased to 15 mT and again stopped at 4 Hz (Figure 4C). When the field was further increased to 20 mT, the devices moved also at 4 Hz (Figure 4C). In analogy with what was done for the data in Figure 4B, the radius of the double bilayer-coated device was evaluated. It resulted to be equal to 350 μm, which is a value considerably higher than that of the single bilayer-coated device. Also in this case, the radius measured is not representative of the thickness of the hydrogel in rest conditions. Indeed, the apparent hydrogel thickness is reduced by its deformation under the device (Figure 4A). To support these observations, optical microscopy (OM) images of the hydrogel layers were acquired and used to determine the mean thickness of the layer. Single bilayer-coated CHT/ALG devices presented a hydrogel global thickness between 200 and 300 μm (Supplementary figure S9). Double bilayer coating, on the contrary, was characterized by highly variable thickness (Supplementary figure S10), resulting from their ellipsoidal shape, between few hundred micrometers and more than 900 μm (in the central region). These values, if compared to the values obtained from the fitting previously operated, support the idea that the hydrogel is somewhat deformed under the device.

Considering the results obtained, the effect of a thick hydrogel layer on the actuability of the devices can be discussed. Apparently, a relevant adhesion force is present between the hydrogel and the glass substrate. Such force needs to be overcome to start device motion, and low magnetic field strengths are not sufficient to generate enough torque to move the device. When the device moves, the interaction significantly alters the linearity of the motion. Devices coated with a double bilayer are highly nonlinear and present a tendency to stop at high actuation frequencies. This is indicative of a larger contact area between the device and the glass substrate. As evidenced in our previous works, all these effects can be reasonably attributed to the presence of adhesion between the glass and the hydrogel rather than to the mass loaded on the device (Bernasconi et al., 2019; Bernasconi et al., 2021).

Another important effect of the hydrogel layer is its ellipsoidal shape. Even if the problem is slightly mitigated by the deformation under the device, the hydrogel is not perfectly cylindrical. This results in a slight deviation from the desired direction along which the devices were moved. In the case of actuation along the y-axis, the deviations reported in Figure 4D were recorded. As expectable, devices coated with a double bilayer were characterized by larger deviations. Bearing in mind all the considerations previously exposed, devices could still be efficiently actuated in a controlled way. By modifying the axis of the rotating field, the direction of the devices was controlled. Figure 4E and Supplementary Video S2 show the circular actuation of a CHT/ALG-coated device, which was obtained continuously by varying the angle of the axis from 0° to 360°. Figure 4F and Supplementary Video S3 depict a zig-zag path, obtained by switching the angle from 0° to 90° and then back to 0°. The ability of the devices to move on inclined planes was verified as well. Figure 4G and Supplementary Video S4 depict the successful actuation of a PAH/ALG device along an inclined plane (characterized by a height of 1 cm, a length of 3 cm, and an angle equal to 18.43°).

## Conclusion

The experimentation carried out for the present work demonstrated the feasibility of multilayer microsystem fabrication following a layer-by-layer approach on magnetically guided microdevices. The latter were successfully microfabricated using stereolithography 3D printing and wet-coated with a sequence of metallic layers, namely copper and CoNiP. As top coating, gold was deposited to ensure chemical inertness and biocompatibility. Gold-coated microdevices were successfully coated with self-assembled layers of positively and negatively charged hydrogels. The introduction of multilayers on the original alginate layer enhanced drug release control by increasing the diffusion path for the drug itself. Release tests evidenced a remarkable decrease in the initial burst release and, in general, a decrease in released amounts at constant time. The presence of thick hydrogel layers induced an interesting effect on the actuability of the devices. Microrobots coated with many hydrogel layers were found to have an ellipsoidal shape and a notable thickness in their central region. The consequence of this shape was a more difficult actuation with respect to their uncoated counterparts. In particular, higher magnetic fields were required to overcome the adhesion force present between the glass substrate and the hydrogel. The motion of the devices was found to be highly nonlinear, with significant directional deviations. Nevertheless, devices were successfully actuated in a controlled fashion. Considering the results obtained, it is reasonable to state that hydrogel multilayer-coated microdevices may be potential candidates to perform targeted drug delivery in some of the large cavities inside the human body, e.g., the digestive apparatus.

## Data Availability

The raw data supporting the conclusions of this article will be made available by the authors, without undue reservation.
