# Supplementary material for: Layer-by-Layer Fabrication of Hydrogel Microsystems for Controlled Drug Delivery From Untethered Microrobots
Source: Front Bioeng Biotechnol. 2021 Oct 13;9:692648. doi: 10.3389/fbioe.2021.692648 (PMC8548779; doi:10.3389/fbioe.2021.692648)
Supplement: Supplementary file 2 [file DataSheet1.PDF]

Supplementary Material for:

Layer-by layer fabrication of hydrogels microsystems for controlled drug delivery from untethered microrobots

Roberto Bernasconi[a], Fabio Pizzetti[a], Arianna Rossetti[a], Brendan Butler[b], Marinella Levi[c], Salvador Pané[d], Filippo Rossi[a,], Luca Magagnin[a,*]*

[a] Department of Chemistry, Materials and Chemical Engineering"Giulio Natta", Politecnico di Milano, via Mancinelli 7, 20131 Milano, Italy

[b] Department of Chemical Engineering, University of Washington, Seattle, Washington, 98195

[c] Department of Chemistry, Materials and Chemical Engineering"Giulio Natta", Politecnico di Milano, piazza Leonardo da Vinci 32, 20133 Milano, Italy

[d] Multi-Scale Robotics Laboratory, Institute of Robotics and Intelligent Systems, ETH Zurich, Tannenstrasse 3, 8092 Zurich, Switzerland

∗ Corresponding authors: filippo.rossi@polimi.it; luca.magagnin@polimi.it

Figure S1 details the dimensions of the microdevices as designed in the CAD file used for the 3D printing step.

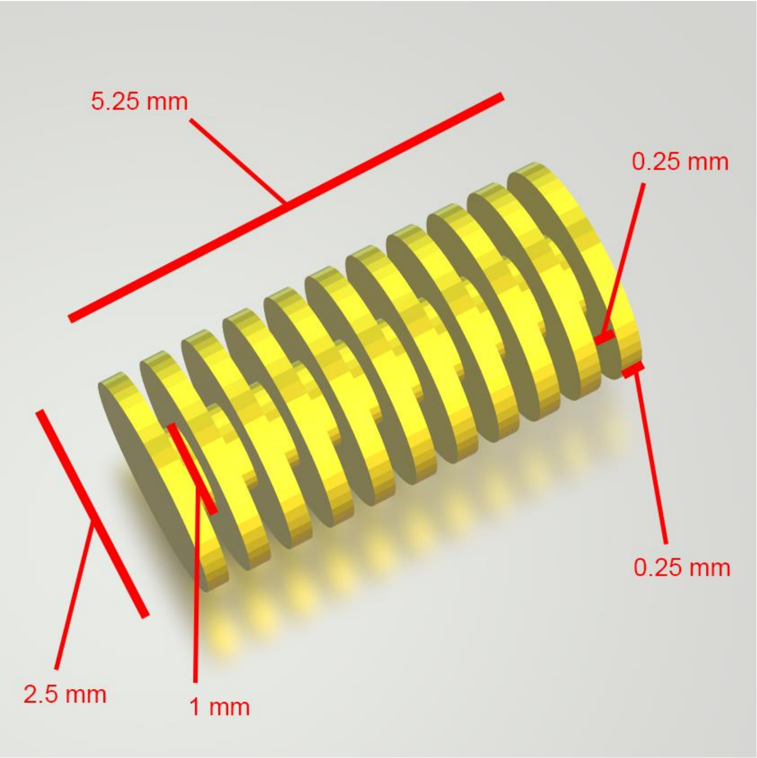

Figure S1. Microdevices theoretical dimensions.

Figure S2 shows the shape of the holder employed to coat the devices with the different hydrogel layers. Devices are hung to a nylon wire that is fixed in the groove visible in figure S2.

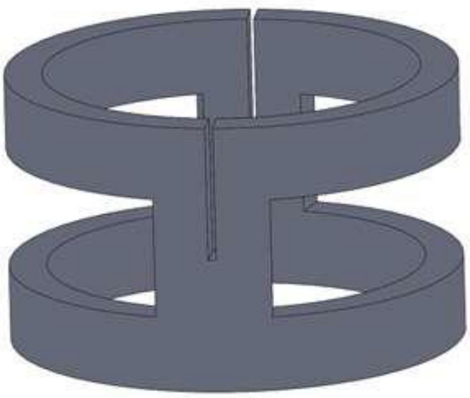

Figure S2. Microdevices holder structure.

Figures S3 and S4 depicts the weight variation observed when alginate coated devices were immersed in sodium citrate at different concentrations (1, 5, 10, 50 and 100 mM). Two different alginate concentrations were used, namely 1 % m/v (figure S3) and 5 % m/v (figure S4).

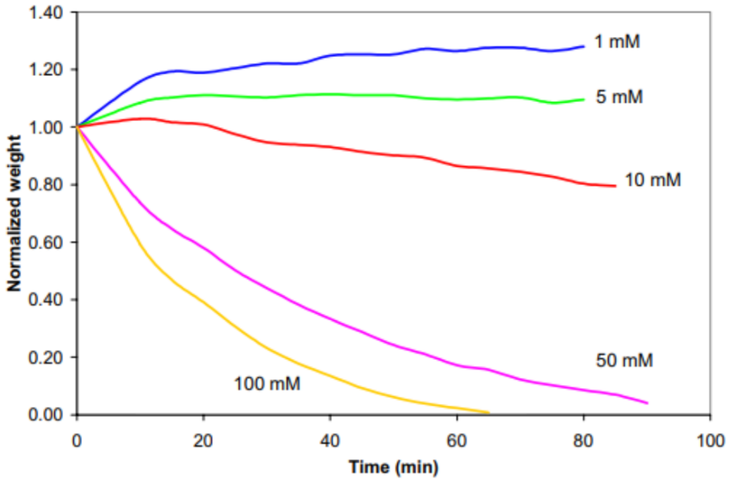

Figure S3. Weight variation for samples coated with alginate from a 1 % w/v solution and immersed in sodium citrate.

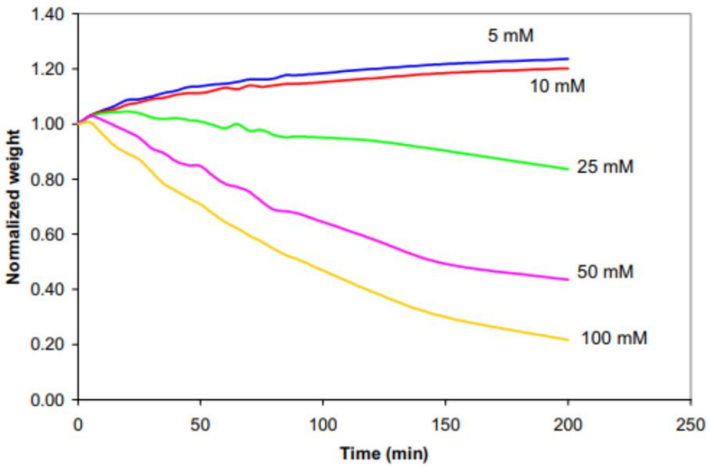

Figure S4. Weight variation for samples coated with alginate from a 5 % w/v solution and immersed in sodium citrate.

Figure S5 shows the result obtained from the EDS characterization performed on a gold coated microdevice.

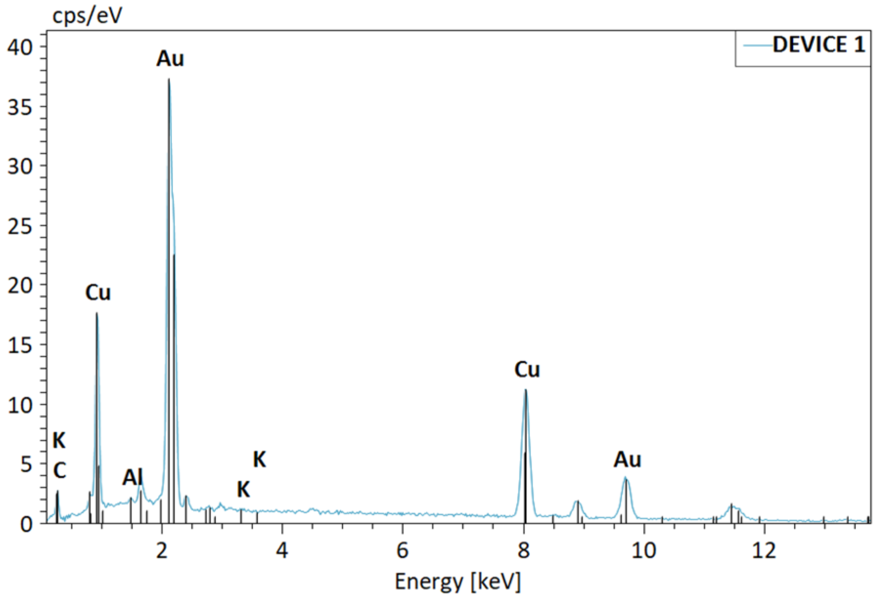

Figure S5. EDS of a gold coated microdevice.

Figures S6, S7 and S8 depicts the chemical structures of alginate, chitosan and poly(allylamine) hydrochloride respectively.

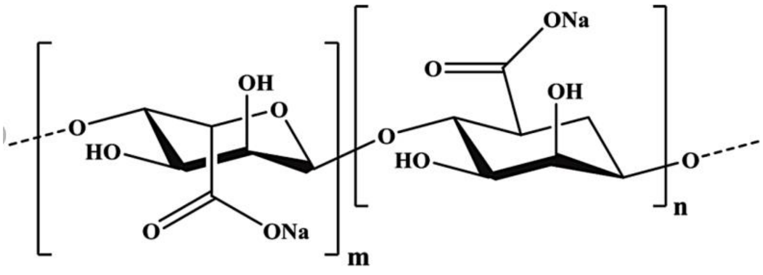

Figure S6. Chemical structure of alginate.

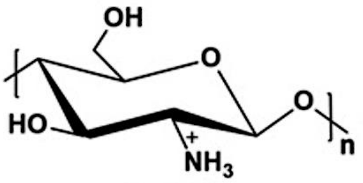

Figure S7. Chemical structure of chitosan.

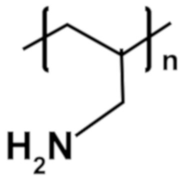

Figure S8. Chemical structure of poly(allylamine) hydrochloride.

Figure S9 depicts the optical microscope (OM) image acquired from a single bilayer coated CHT/ALG device. The external thickness of the hydrogel layer was determined analyzing the image.

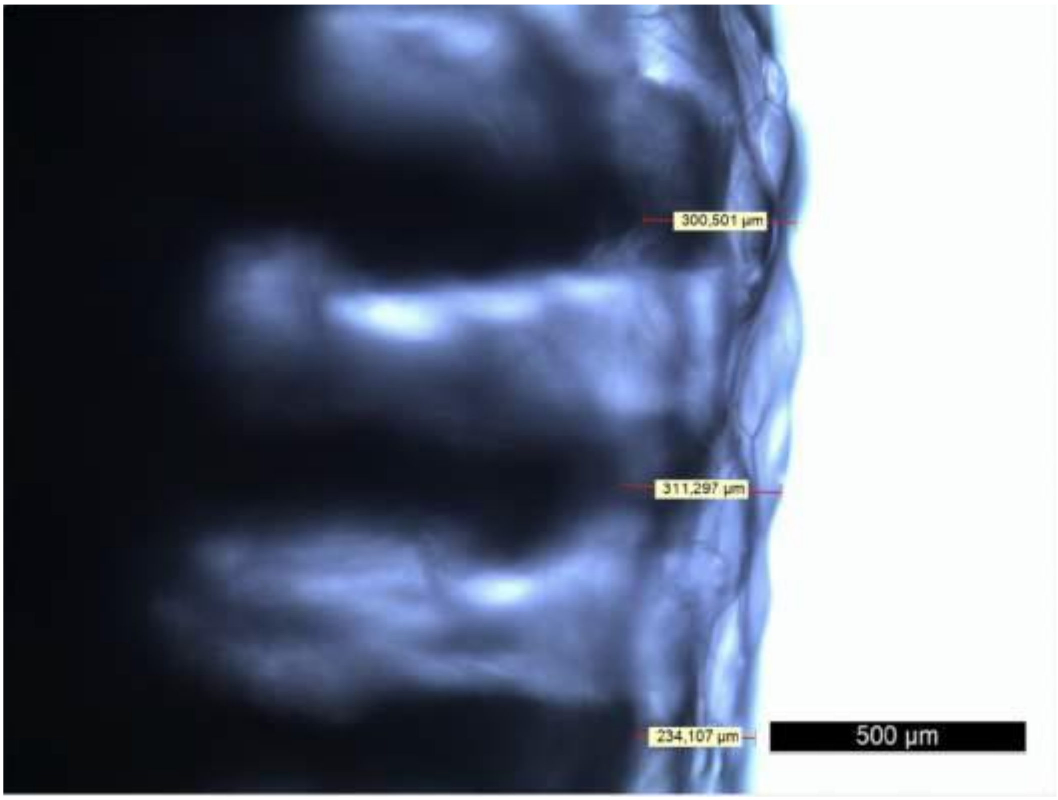

Figure S9. OM image of a single bilayer coated device.

Figure S10 depicts the OM image acquired from a double bilayer coated CHT/ALG device. The external thickness of the hydrogel layer was determined analyzing the image.

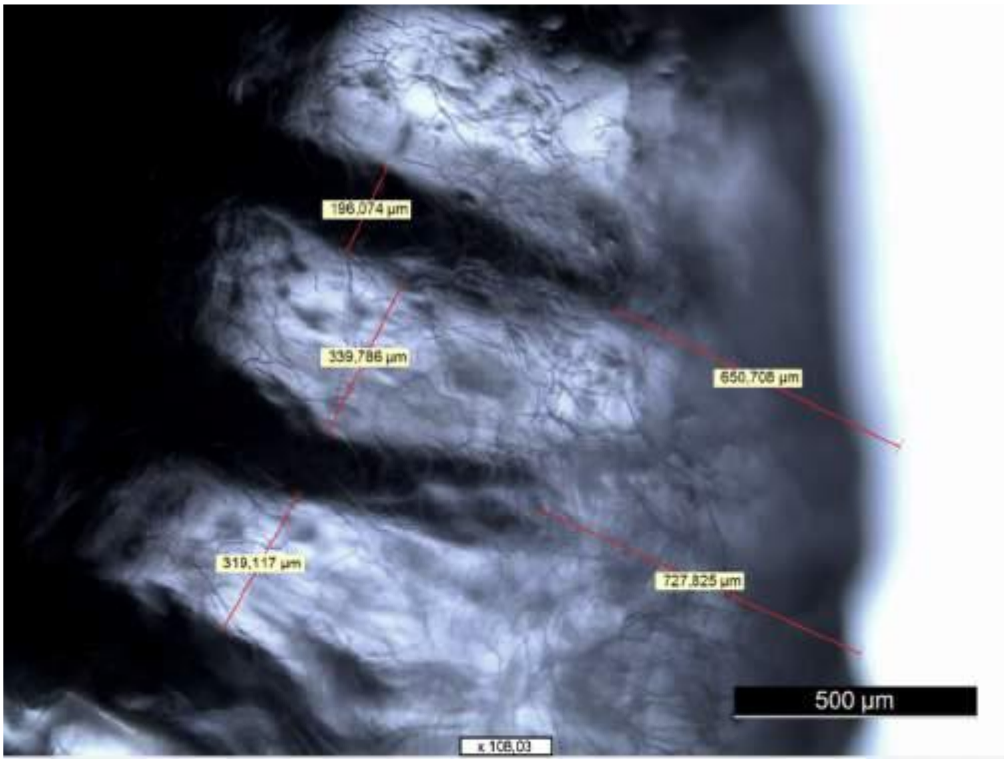

Figure S10. OM image of a double bilayer coated device.
